# Telerehabilitation: Vestibular Physiotherapy vs. Multicomponent Exercise for Functional Improvement in Older Adults: Randomized Clinical Trial

**DOI:** 10.3390/jcm13144279

**Published:** 2024-07-22

**Authors:** Marina López-García, José Jesús Jiménez-Rejano, Carmen María Suárez-Serrano

**Affiliations:** Department of Physiotherapy, Faculty of Nursing, Physiotherapy and Podiatry, University of Seville, 41009 Seville, Spain; jjjimenez@us.es (J.J.J.-R.); csuarez@us.es (C.M.S.-S.)

**Keywords:** telerehabilitation, aging, physical performance, physiotherapy, vestibular exercise, vestibular rehabilitation

## Abstract

The loss of vestibular and motor function can occur naturally with aging. Vestibular physiotherapy exercises (VE) specifically address vestibular rehabilitation to reduce imbalances and improve physical condition, like therapeutic exercise (TE). During COVID-19, technology was used with the elderly for implementation. **Objective:** to determine if vestibular exercises are as effective as multicomponent exercises in improving functional capacity using technological tools. **Methods:** A randomized clinical trial compared two intervention groups of 21 subjects with functional limitations and frailty (average age 76.11 years). The intervention involved multicomponent exercise for the control group (Vivifrail protocol) and vestibular exercises for the experimental group (Cawthorne and Cooksey exercises) for 6 weeks with five sessions per week both groups. Two professionals implemented the exercises, and participants received tailored exercise videos. Primary outcomes were gait speed, dynamic balance, and physical capacity. **Results**: Both groups showed significant improvements. For physical function measured by SPPB (0–12 points), the multicomponent exercise group improved by 1.97 (0.91; 3.03), *p* < 0.001, and the vestibular exercise group improved by 1.63 (0.65; 2.60), *p* = 0.002. For dynamic balance measured by the Timed Up and Go (TUG) test, the multicomponent exercise group improved by −0.88 (−1.33; −0.42), *p* < 0.001, and the vestibular exercise group improved by −0.79 (−1.21; −0.37), *p* < 0.001. There were no significant differences between groups. Finally, regarding gait speed, there were no differences in either group between pretest and post-test (*p* > *0*.05). **Conclusions:** Both multicomponent exercise and vestibular exercises improve functional capacities via telerehabilitation as measured by the SPPB, although vestibular physiotherapy does not show superior outcomes compared to the control group.

## 1. Introduction

Aging is a normal process characterized by gradual changes in the physiological functions of various systems, and within these processes, the loss of vestibular function can produce symptoms such as dizziness, imbalances, facial and limb weakness, confusion, or headaches [1,2,3]. To highlight the prevalence of these problems, we had to know that approximately 15% of adults over the age of 60 experience some form of functional decline, and this percentage increases with age, affecting more than 30% of individuals over the age of 70. In fact, sarcopenia, in addition to the loss of muscle mass, strength, and function, also includes weakness, fatigue, balance problems, and difficulties in walking and standing. Both sarcopenia and pre-vestibulopathy increase the risk of falls, hospitalizations, and a decrease in quality of life [4].

Considering the demographic transformation that Spain is undergoing, with 14.4% of the population being over 70 years old, we can say that aging has become a problem impacting public health [5].

Vestibular physiotherapy has been shown to be effective in improving balance and reducing the risk of falls in cognitively intact individuals without vestibular impairment. The exercises, specifically designed for vestibular rehabilitation, help reduce dizziness and imbalances by facilitating central nervous system compensation [6,7]. Since the 1990s, the main contributions in this area of knowledge have been the exercises proposed by Cawthorne and Cooksey, which have demonstrated effectiveness. These simple exercises involve the head and eyes and are used in all types of patients with nonspecific symptoms and without pathological findings [8].

However, given that body balance control in the elderly depends not only on the vestibular system but also on the interactions between all other systems, it seems interesting to add exercises with multiple components [9]. This could improve the functional independence of the elderly for daily activities and body balance control. For decades, the improvement in the physical condition of older adults through the reduction of sarcopenia via exercise has been researched and proven. The trend in research suggests that this loss of strength and function appears to be reversed (with moderate to high evidence) through resistance exercises combined with aerobic and balance training, improving quality of life, and enhancing gait speed through the combination of resistance and balance exercises [10]. Clinical Practice Guidelines emphasize the importance of multicomponent exercise (including strength, cardiovascular training, flexibility, and balance) for this population group. The Spanish Ministry of Health and Social Services has issued a consensus on frailty prevention in the National Health System with a specific guide for multicomponent exercise as a resource to be developed in the community [11].

During the COVID-19 pandemic, physiotherapists transitioned to providing telehealth services and recognized it as an effective platform that generated similar health outcomes [12,13].

Therefore, given the evidence of the independent use of both interventions in the elderly, it is necessary as an objective to determine if there is a difference in the efficacy of vestibular exercises compared to multicomponent exercises for improving functional capacity when applied in a setting, after evaluation, using technological tools to provide health care. The main hypothesis is that vestibular exercises are more effective than multicomponent exercises in improving functional capacity in individuals over 70 years old.

## 2. Materials and Methods

Design: Controlled and randomized clinical trial (RCT) with two parallel groups, comparing a protocol of vestibular exercises (VE) and a control group with multicomponent exercises (ME) in individuals over 70 years old. The design of this study follows the CONSORT guidelines [14].

The trial was registered at www.clinicaltrials.gov, (accessed on 20 May 2019) with registration number NCT04894929. The study obtained the approval of the Biomedical Research Committe Biomedica of Virgen de Valme Hospital with registration number 1853-N-18, 28 April 2022. Additionally, the study protocol adheres to the ethical standards and guidelines established by the Declaration of Helsinki.

Participant Selection Criteria:

- Women and men over 70 years old.
- Subjects identified with functional limitations and a high probability of frailty through the Short Physical Performance Battery (SPPB), scoring between 4 and 9 points [13,14,15,16].

Exclusion Criteria:

- Patients who did not have independent walking or who obtained scores above 10 or below 4 in the previous SPPB evaluation.
- Polypharmacy patients (combined use of beta-blockers, sulpiride, or betahistine).

Recruitment: Patients over 70 years old who voluntarily attended physiotherapy at Los Bermejales Physiotherapy Clinic, Seville (Spain), where the pre- and post-evaluations took place.

**Figure 1:**
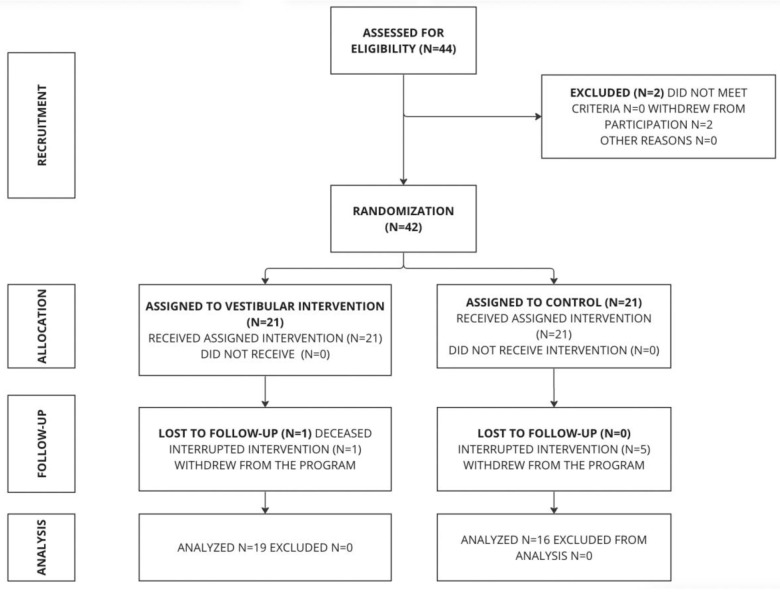
Study flow diagram according to CONSORT.

Intervention: An information session was conducted at the physiotherapy center for participants (*n* = 44). They were evaluated (the evaluation using SPPB confirmed that the patients were in a frail or pre-frail state, allowing us to assign the exercises appropriate to their functional status) and provided written informed consent. Eligible participants were randomized into the vestibular exercise group and the multicomponent control group (Figure 1).

Study Variables and Measurement Instruments Dependent Variables:

(1) Physical function assessment: Using the SPPB scale, a discrete quantitative variable (0–12 points). The SPPB includes balance assessment, 4 m walk test, and the Time Up and Go (TUG) test. All of them demonstrate that the Short Physical Performance Battery (SPPB) is a valid and reliable measure for assessing physical performance in older adults with mild cognitive impairment (MCI). Intraclass Correlation Coefficients (ICC) above 0.7 indicate sufficient reliability for group comparisons. Additionally, SPPB subcomponents, such as the 4 m walk time (4mwt), show high reliability (ICC > 0.9), making them suitable for individual measurements over time. The Minimal Detectable Change (MDC) values provide thresholds for detecting real changes in performance, facilitating better monitoring and intervention planning. This confirms that the SPPB and its subcomponents are reliable tools for evaluating and monitoring physical performance in this population [15].
  - Classification: Severe limitation D (0–3 points), moderate/frail C (4–6 points), mild/pre-frail B (7–9 points), minimal limitation A (10–12 points).
(2) Gait capacity: Authors such as O’Hoski et al. (2020) discussed the significant validity of physical function tests conducted over distances of 6 and 4 m (as we have developed), which have scientific evidence and are more feasible due to performance and space considerations. O’Hoski et al. evaluated physical function and frailty to predict adverse outcomes in older adults, confirming the validity of these tests in different configurations and their utility in clinical practice to measure dynamic balance and fall risk. Gait capacity, using the 4 m walk test, is a continuous quantitative variable measured in meters per second (m/s). A high probability of frailty is indicated by a gait speed of less than 0.8 m/s [15,16].
(3) Dynamic balance capacity as a predictor of fall risk: Using the TUG test, a continuous quantitative variable measured in seconds. The national document uses a cutoff of ≥20 s for high frailty probability, but >12 s is more aligned with current literature. Among the studies that most reliably demonstrate the TUG’s effectiveness in measuring dynamic balance is the study by Barry et al. (2014), a systematic review and meta-analysis that identified the TUG as a useful predictor of fall risk in community-dwelling older adults, highlighting its utility in clinical practice to assess functional mobility and dependency risk. This study underscores the validity and reliability of the TUG, confirming its consistency and accuracy in evaluating the functional capacity of older adults [15,16,17,18,19].
(4) Sociodemographic Variables: Age (continuous quantitative variable measured in years) and sex (nominal dichotomous qualitative variable: Male/Female).

Intervention and randomization: An information session was conducted at the physiotherapy clinic for interested patients (n = 44). Participants were evaluated (the evaluation using SPPB confirmed that the patients were in a frail or pre-frail state, allowing us to assign the exercises appropriate to their functional status) and provided written informed consent.

The intervention was divided into two groups: an experimental vestibular exercise group and a multicomponent exercise control group. Participants were randomly assigned, by simple randomization method using the Python program, to either the multicomponent home exercise group (control group: 16 subjects) or the vestibular exercise group (experimental group: 19 patients) as detailed in the flow diagram.

The intervention involved 5 sessions per week for 6 weeks. Two expert physiotherapists implemented the exercises and created tailored exercise videos. Exercises were sent via email by an administrative staff member who did not participate in the evaluations. The administrator phoned the participants weekly to verify their adherence, requesting them to complete at least 90% of the sessions for re-evaluation.

**Figure 2:**
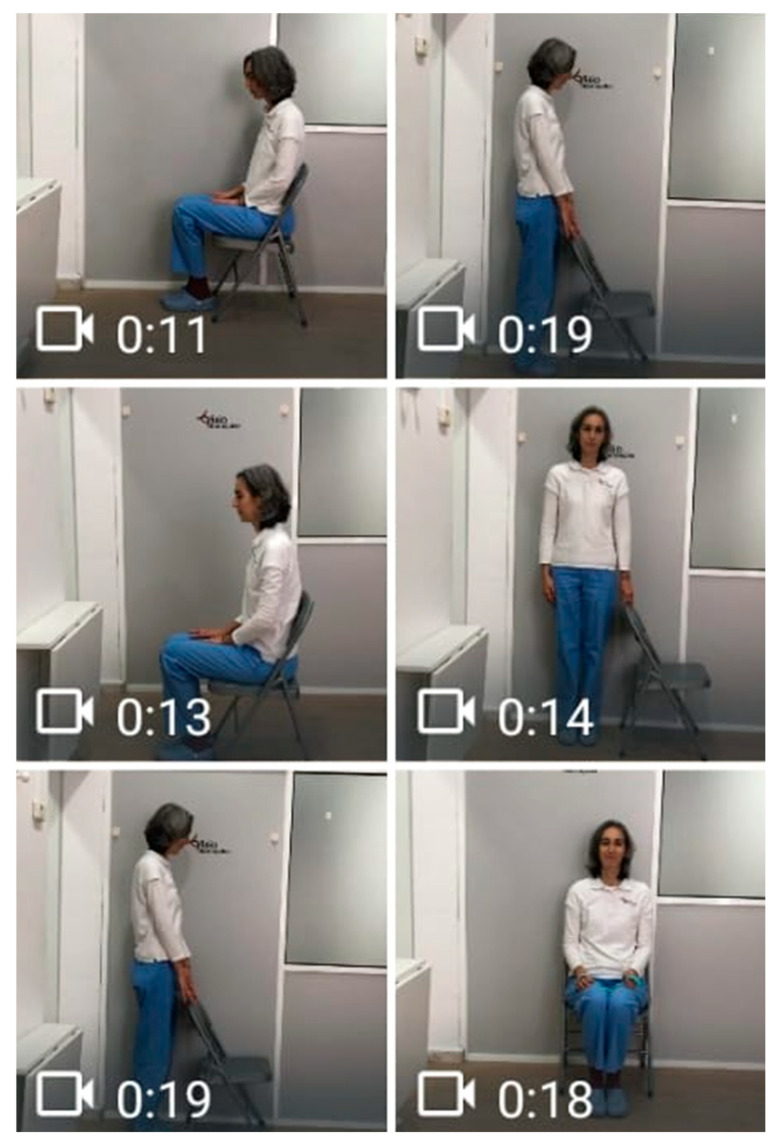
Examples of videos sent to patients for multicomponent exercise.

The interventions were:

A. Multicomponent Home Exercise Control Group: A 6-week therapeutic multicomponent exercise program following the ministerial guide and Vivifrail consensus (Table 1). Patients received exercise videos via email and committed to 5 weekly sessions (Monday to Friday), each lasting approximately 45 min. The order and frequency of each of the exercise components performed by the participants, including cardiovascular, balance, strength, and flexibility exercises, are determined in the guide itself (Figure 2).

- Strength exercises: seated arm flexion and extension with weights, seated arm flexion and extension with elastic band, hand grip exercises seated horizontal arm opening with elastic band, seated diagonal arm opening with elastic band, seated knee extension without weight, standing calf muscle exercise, hip separation exercise, hamstring exercise, leg flexion without chair.
- Balance exercises: Walking in line, one-leg balance with crossed arms, toe-heel balance exercise, walking on toes and heels with support.
- Stretching exercises: Thigh muscle stretch, hamstring stretch, arm muscle stretch, lateral neck muscle stretch, seated ankle flexibility exercise, neck muscle stretch, arm stretch.
- Cardiovascular (aerobic) exercises: walk 12–15 min.

**Figure 3:**
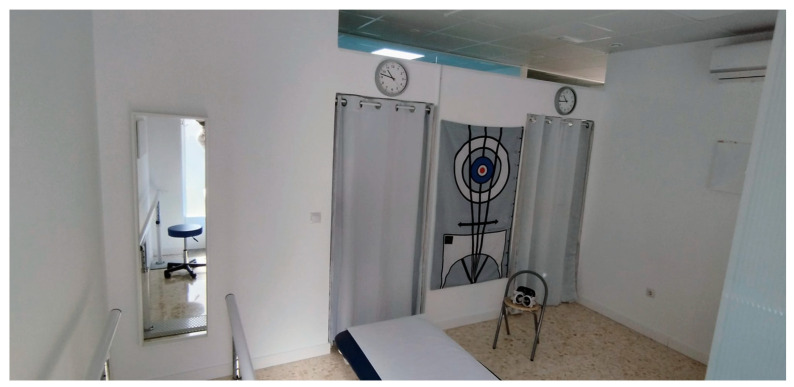
Space prepared for the instructions and recording of vestibular exercises.

B. Home Vestibular Exercise Group: Vestibular exercises were performed under the supervision of a physiotherapist on the first day to learn them, with session durations of about 20 min and 5 sessions per week, proposed by Cawthorne and Cooksey [8]. The exercises were later recorded and sent via email to the patient for home practice (Figure 3). The exercises consisted of 15 repetitions without fatigue of the following:

a. Eye movements while sitting and standing.

b. Head and body movements while sitting and standing (Vorx1 and VorX2).

c. Standing visual tracking exercises: Hold a business card (with text lines) at eye level about 30 cm away. Slowly move the card left and right, up and down, and diagonally. Keep your head still and follow the card’s movement with your eyes, always keeping the text in focus. Repeat the exercise 10 to 15 times in each direction. As you improve, increase the speed and range of card movement, always keeping the text in focus, and gradually decrease the size of the letters on the card until you can barely read the text.

d. Combined exercises involving step modifications, unstable surfaces, and walking.

Follow-up: All participants, both in the control and experimental groups, kept a diary from Monday to Friday to monitor attendance and compliance. There were ultimately 30 sessions, with 7 dropouts due to nonadherence to the exercises.

Blinding: Participants, the post-intervention evaluator, and the data analyst were blinded to the intervention to which the subjects had been assigned.

Sample size calculation: For the calculation of the sample size, the GPower 3.1.9.7^®^ software was used. Data from the study conducted by Jofré-Saldía et al. [19] were used, where the functional capacity variable measured with the SPPB questionnaire in the experimental group obtained a post-intervention value of 11.80 points (SD = 0.47) and in the control group, 9.10 (2.40). An α error of 0.05 was considered with a study power of 80%, two groups, one-tailed hypothesis, and a proportion between groups of 1. With these data, a sample of 22 subjects was obtained, 11 in each group. A possible dropout rate of 15% was estimated, raising the sample to 26 subjects (13 in each group). Finally, 42 subjects were included, 21 in each group, with 2 dropouts in the experimental group, leaving 19 subjects, and 5 dropouts in the control group, leaving 16 subjects.

Data Analysis: For data analysis, the Statistical Package for the Social Sciences (SPSS) program, version 29.0 for Windows, will be used. A descriptive analysis was performed, calculating absolute frequencies and percentages for nominal variables, while for quantitative variables, the mean and standard deviation were provided when normally distributed. For quantitative variables that did not follow a normal distribution, the median and first and third quartiles (Q1–Q3) are shown. Initial homogeneity between the experimental and control groups was then studied concerning sex, age, and pretest values of all dependent variables. Subsequently, a mixed factorial ANOVA was used for the physical function and dynamic balance variables. For gait capacity, various intragroup measurements were compared. Finally, to compare intergroup gait capacity, the difference between pre-treatment and post-intervention values was calculated, referred to as “Difference in scores.” Additionally, the percentage change in scores between pretest and post-test was calculated using the formula:

Percentage change in scores = (Post-test − Pretest) ∗ 100/Pretest

The values obtained in the post-test measurement of gait capacity, the “Difference in scores”, and the “Percentage change in scores” were compared, and the effect size was estimated. A “per protocol” analysis was performed to study the effects of the applied intervention. All statistical tests were performed considering a 95% confidence interval (CI) (*p*-value < 0.05).

## 3. Results

A total of 42 subjects were included in the study, with seven dropouts: two subjects from the multicomponent exercise (ME) group and five from the vestibular exercise (VE) group, leaving a final total of 35 subjects (16 in the ME group and 19 in the VE group). Table 2 shows the initial characteristics of the subjects and the pre-intervention values of the dependent variables. No significant differences were found between the groups in any of the mentioned characteristics and dependent variables (Table 2).

### 3.1. Dynamic Balance and Physical Function

#### Outcomes

**Figure 4:**
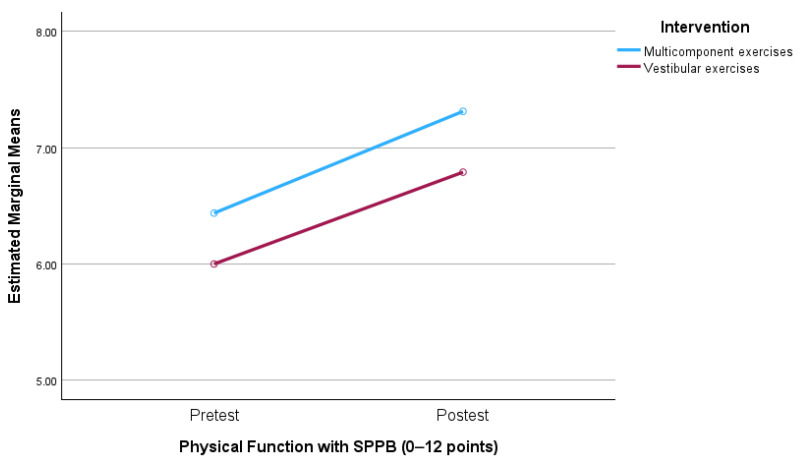
Outcomes in physical function. (0–4 points) means dependent, moderate/frail C (4–6 points), mild/pre-frail B (7–9 points) and with minimal limitation A (10–12 points).

**Figure 5:**
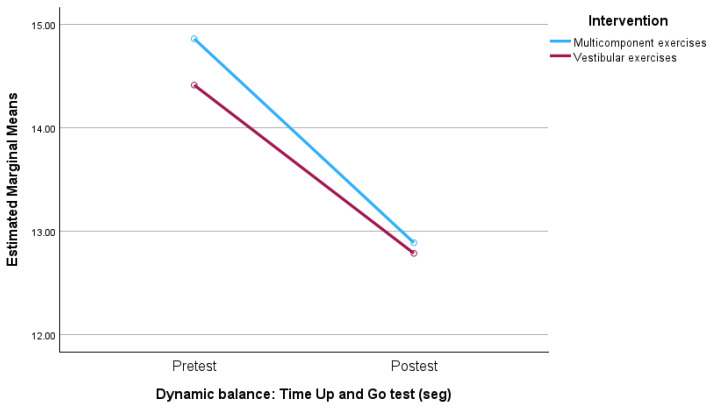
Outcome in dynamic balance.

- The mixed ANOVA analysis of the Physical Function and Dynamic Balance variables did not show a significant interaction between the time factor and the treatment, nor a significant effect of the between-subjects factor.
- There was a significant difference in the within-subjects factor (Table 2). In other words, although both groups significantly improved in both variables, there were no significant differences between the two groups in any of the measurements carried out on these variables (Figure 4 and Figure 5).

### 3.2. Dependent Variables

Intragroup Comparisons of the Results Obtained in the Dependent Variables Studied Two Groups (Table 3).

**Figure 6:**
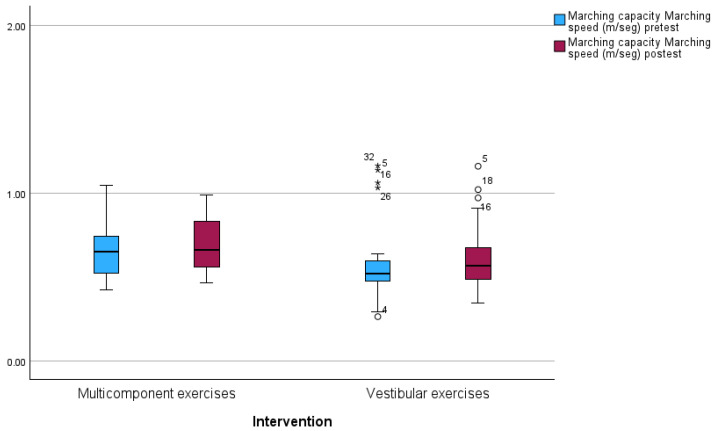
Comparison ME vs. VE. Box plot of the two measurements of gait capacity performed in the two treatment groups. ○: Outlier (between 1.5xInterquartile range and 3xInterquartile range). *: outilier > 3xInterquartile range).

- We present the results of the intragroup comparisons of the variable gait capacity, considering each group separately. In neither of the two groups were there significant differences between pretest and posttest (Table 4).

- The results suggest that there are no statistically significant differences in the measurements of gait capacity and speed (m/s) after the multicomponent exercise and vestibular exercise interventions (Figure 6). The high *p*-values and small effect sizes indicate that the interventions did not have a considerable impact on the measures of the studied variables (Table 5).

## 4. Discussion

In the clinical trial conducted, no significant differences were found between the improvements produced by vestibular and multicomponent exercises. Both the control and experimental groups showed improvement in performance tests following their respective interventions. The difference in the total SPPB score leans toward a more significant improvement. Both EV and EM aim to combat inactivity and deconditioning. Inactivity underlies cardiovascular deterioration, insulin resistance, depression, and notably musculoskeletal disorders like sarcopenia [20,21,22]. The European Consensus review suggests overlap in these processes, indicating that balance disorders can lead to low muscle strength, as evidenced in our results. Additionally, the consensus confirms that physical performance is linked to locomotion, involving muscles as well as central and peripheral nervous functions, including balance [23]. This may lead us to think that, at times, physical condition may deteriorate and lead to a decline in physical performance, balance, and gait tests, while in other instances, presbivestipulopathy associated with aging may be the cause of this decrease. It is worth noting that other authors such as Velez et al. [24] have already established that the design, following a similar study using home-based telerehabilitation guided by exercises, emerges as a viable strategy to enhance functional capacities in older adults. This study highlights key aspects influencing the successful implementation of home-based rehabilitation and telerehabilitation services. They examined 223 studies and analyzed 53 in depth. The findings suggest that both in-person and telerehabilitation services are perceived as convenient and less disruptive to daily activities, especially in the COVID-19 context. Additionally, these services can promote self-management and empower patients. The review indicates that well-guided telerehabilitation, complemented by in-person sessions, is a promising strategy for improving functional capacities in older adults. The author also established that home-based telerehabilitation guided by exercises is a viable strategy for enhancing functional capacities in older adults.

In individuals over 70 years of age, the frequent overlap of pathological conditions poses an evaluation challenge for the cause of symptoms described as dizziness or instability. Some research [24,25] agrees that in addition to evaluating vestibular function, physical examination should also assess the musculoskeletal system, as they are closely related to sarcopenia. Therefore, functional performance measures are becoming highly reliable and preferred indicators for assessing health and function in older adults, and evidence has been supporting their use as measures of change for years [25,26]. Therefore, in our study, we used European Consensus tests such as the Short Physical Performance Battery (SPPB), the Timed Up and Go (TUG) test, and gait speed to measure these variables. These tests are not only indicative of sarcopenia but also assess balance and mobility, providing a comprehensive view of the patient’s physical state and their risk of falls and other adverse outcomes [23].

The set of variables has been taken with reference tests and has broad support for its usefulness. Several studies have assessed the usefulness of the SPPB for evaluation with significant modifications [27] through scheduled exercise for therapeutic purposes, as well as various exercise programs, and the conclusion is that it allows for showing changes and being sensitive to adverse effects such as hospital admissions and a predictor of falls, making it a useful measure even in the medium term [10,28]. Furthermore, in the case of institutionalized settings, physical training alone does not seem to be sufficient to reduce and prevent falls, although there is controversy as to whether exercise improves more or not when supervised [29,30].

The studies conducted by Latham et al. [29] with a population similar to our study (but with hip fracture and 6 months of follow-up) saw the clinically important difference on the SPPB scale was 0.3 to 0.8 points, and with respect to the moderate effect size of 0.4 (equivalent to moving from 6 to 7 on the score). These changes clearly determine the jump according to the test itself from a functionally higher type B to C. That is, to improve and go from moderate limitation or a frail patient (who presents with difficulty walking or with assistance, who gets up and completes balance tests with difficulty) to being a pre-frail patient (whose gait is already autonomous, although it retains gait disorders, with difficulty in doing five stand-ups and a subtle balance alteration) [16]. In our study, both the ME and VE groups obtained scores exceeding this figure, which reaffirms us in the usefulness of the designed exercises. Other studies [16,29,30,31] (conducted with a younger sample and composed of more individuals) point in the same direction, with a functional level situated at C, so the sensitivity margin is narrower [28], although it still marked a point as a limit [30]. It sems that the inflection point is marked close to this point, with some authors establishing that a score of less than 8 implies severe mobility disability and even anticipate an 82% probability of developing falls in a 2-year follow-up.

Studies [24,32] have shown that telerehabilitation is effective for treating vestibular disorders, improving gait stability and balance during the COVID-19 pandemic. The systematic review made by Kundakci [33] included individuals over 18 years old, not specifically older adults. These studies compared vestibular rehabilitation exercises to placebo or other interventions and concluded that the exercises improved balance capacity as per the finally selected articles. The findings of this research support the idea that vestibular telerehabilitation can be a useful strategy to improve balance and daily functionality in people with vestibular disorders, thus emphasizing its potential benefit in this specific area [12].

This may indicate, as many authors comment, the need for complete and transdisciplinary work [29], although much progress is needed in the standardization of exercises [29] and in evidence of their usefulness in reducing falls. Regarding the suitability of both techniques and their measurement according to TUG, our study has shown improvements in both groups, although there are authors [33] who differentiate performing VE in subjects with reduced stability limits with great improvement and patients with more time in the TUG show worse results and can benefit more from gait training. In this sense, a systematic review [33] also suggests that VR with exercises shows benefits for adult patients with chronic dizziness with respect to improvement in the vertigo symptom scale, risk of falls, and balance, without reaching an association like other authors with reductions of up to 50% in risk of falls with significant reductions in TUG. It may lead to the intervention period of only 6 weeks being understood as a limitation, even if they are daily sessions.

Therefore, the interventions seem to indicate that they improve physical condition, although our study cannot infer a reduction in the risk of falls. Many authors find themselves in the same dilemma, justifying post-test improvements to the intervention in subacute contexts [29] and even suggesting ultimately that exercise programs should be combined multicomponent (balance-coordination, strengthening and aerobic exercises) to improve balance, postural control, cognition, muscle strength, and quality of life and that specific programs for older adults involving moderate to high and challenging balance tasks could reduce the rate of falls. Perhaps the usefulness of technology-based programs can be suspected, but other authors [32] already raised the degree of acceptance and adherence of older adults to technology-based exercise interventions, making a systematic review that included more than 20 studies in this population comparing traditional face-to-face programs with technology-based exercise programs, finding high adherence in both types of interventions (even adherence was higher in technology-based interventions, regardless of study location, level of supervision, and mode of delivery). As we pointed out in our study, and these authors also conclude, the exercise interventions were supervised. The review highlights that technology offers an acceptable way to provide older adults with attractive exercise opportunities, with high adherence rates in both supervised and unsupervised settings for at least the first 12 weeks of the intervention (considering that our intervention is 6 weeks although Vivifrail marks up to 12 weeks for the first re-evaluation). Another good point is that the average age range of the cohort was 67 to 86 years, similar to our study that was 69.55 to 82.67.

In the same line, other studies investigated the positive impact of visual stability and balance exercises through telerehabilitation in individuals with these disorders during the COVID-19 pandemic. These findings, aligned with the research of Harrell et al., suggested that vestibular telerehabilitation can be a useful strategy to improve balance and functionality in the daily life of people with vestibular disorders, highlighting its potential benefit in this area [12].

It is clear, therefore, that it is necessary to identify a patient profile whose improvement in balance directly influences the standardized tests by these new means and methods (and have proven to be useful in detecting frailty, and even fall risk), which are usually treated solely through multicomponent exercise.

Limitations of the study: The difficulty in obtaining the sample to perform the number of daily sessions was a real challenge given the age and adherence presented by populations with these characteristics. Furthermore, we consider as another limitation of our study the impossibility of re-evaluating after 6 months to verify the permanence of the effects, due to the impact of COVID and the resulting restrictive measures, together with the unfortunate loss of the sample for a possible reassessment.

Additionally, as a limitation, it should be noted that the small sample size may affect the validity of the results due to potential heterogeneity.

## 5. Conclusions

There are indications of the efficacy of multicomponent exercise interventions and vestibular exercises to improve functional capacities measured according to SPPB through telerehabilitation, although vestibular physiotherapy does not demonstrate in this study to be superior in functional results to the control group.

Gait speed improves if multicomponent and vestibular exercises are performed, which are also home-based and mediated by technology. Both the vestibular exercise group and the multicomponent exercise group showed significant improvements in gait speed and dynamic balance in older adults through telerehabilitation. No significant differences were found between the two types of interventions in the measurements performed.

Telerehabilitation is a viable and effective tool for functional improvement in older adults, facilitating adherence to exercise programs during situations such as the COVID-19 pandemic.

According to the evidence, new studies are necessary that compare the effectiveness of a group that performs both interventions jointly against other scheduled exercises.

## Figures and Tables

**Table 1:** Multicomponent exercise’s distribution per week.

Patient Type	Strengthening Work (Days/Week)	Flexibility Work (Days/Week)	Aerobic Walking	Balance Work
Pre-frail (Type B)	3	4	5 days a week	5 days a week
Frail (Type C)	3	5	5 days a week	5 days a week

**Table 2:** Initial characteristics of the subjects.

Outcome		Sample Total *n* = 35	ME Group *n* = 16	VE Group *n* = 19	*p* Value Signification
Age (years old), Mean (SD)		76.11 (6.56)	75.25 (6.79)	76.84 (6.45)	*p* = 0.483 ^a^
Sex, *n* (%)	Male	13 (37.1%)	6 (37.5%)	7 (36.8%)	*p* = 0.968 ^b^
	Female	22 (62.9%)	10 (62.5%)	12 (63.2%)	
Physical function with SPPB (0–12 points), Mean (SD)		6.20 (1.53)	6.44 (1.63)	6.00 (1.45)	*p* = 0.795 ^b^
Marching capacity Marching speed (m/s), Median (Q1–Q3)		0.63 (0.23)	0.65 (0.52–0.74) ^c^	0.52 (0.47–0.59) ^c^	*p* = 0.172 ^d^
Dynamic balance: Test Time Up and Go (s), Mean (SD)		14.62 (4.98)	14.86 (4.41)	14.41 (5.52)	*p* = 0.408 ^b^

^a^ Student’s *t*-test for independent simples was used. ^b^ Pearson’s bilateral asymptotic test was used. ^c^ Median and the first and third quartiles are shown. ^d^ Mann-Whitney’s test was used. ME: Multi-modal exercise. VE: Vestibular exercise. Q1–Q3: First and third quartiles. SD: Standard Deviation

**Table 3:** Results of factorial analysis of the variables.

Variable				
Dynamic balance: Test Time Up and Go (s)	Interaction time vs. Treatment	F_(1, 33)_ = 0.24, *p* = 0.629; η_p_^2^ = 0.007		
	Inter-subjects Group Factor	F_(1, 33)_ = 0.03, *p* = 0.863; η_p_^2^ = 0.001		
	Difference of averages in post-test Inter-group and CI	ME	VE	Average diff./*p*-value
		12.89 (4.05)	12.78 (4.81)	0.11 (−2.99; 3.20) *p* = 0.947 d = −0.02
	Intra-subjects Factor	F_(1, 33)_ = 25.88, *p* < 0.001; η_p_^2^ = 0.439		
	Difference of averages Intra-group, CI al 95%	ME	pretest vs. postest	1.97 (0.91; 3.03) *p* < 0.001
		VE	pretest vs. postest	1.63 (0.65; 2.60) *p* = 0.002
Physical function with SPPB (0–12 points)	Interaction time vs. Treatment	F_(1, 33)_ = 0.08, *p* = 0.782; η_p_^2^ = 0.472		
	Inter-subjects Group Factor	F_(1, 33)_ = 0.84, *p* = 0.366; η_p_^2^ = 0.025		
	Difference of averages in post-test Inter-group and CI	ME	VE	Average diff./*p*-value
		7.31 (1.70)	6.79 (1.65)	0.52 (−0.63; 1.68) *p* = 0.364 d = −0.31
	Intrasubjects Factor	F_(1, 33)_ = 29.51, *p* < 0.001; η_p_^2^ = 0.472		
	Difference of averages Intra-group, CI at 95%	ME	pretest vs. postest	−0.88 (−1.33; −0.42) *p* < 0.001
		VE	pretest vs. postest	−0.79 (−1.21; −0.37) *p* < 0.001

SPPB: Short Performance Battery functional capacity test; ηp^2^: Partial squared eta coefficient; d: Cohen’s “d”; CI: Confidence interval. Source: self-elaboration. F: Fisher-Snedecor´s F. ME: Multicomponent exercise. VE: Vestibular exercise.

**Table 4:** Intragroup comparisons of the results obtained in the studied dependent variables.

Variable	Group	Measure Median (Q1–Q3)		Signification
		Pretest	Post-Test	
Marching Capacity Speed (m/s)	ME	0.66 (0.17) ^a^	0.69 (0.17)^a^	*p* = 0.298 ^b^
	VE	0.52 (0.47–0.60)	0.56 (0.49–0.67)	*p* = 0.243 ^c^

^a^ Average and typical deviation are shown. ^b^ Student’s *t*-test was used for related samples. ^c^ Ranges’s test with Wilcoxon sign was used. ME: Multimodal exercising. VE: Vestibular exercising; Q1–Q3: First and third quartiles.

**Table 5:** Intergroup comparisons of the results obtained in the dependent variables.

Variable		Measurement Differences		Significance Effect Size
		Group EM Median (Q1; Q3)	Group EV Median (Q1; Q3)	
Gait Capacity Speed (m/s)	Posttest	0.66 (0.53–0.85)	0.57 (0.49–0.67)	*p* = 0.150 ^a^ r = −0.24
	Difference	−0.02 (−0.04–0.04)	−0.02 (−0.06–0.01)	*p* = 0.691 ^a^ r = −0.07
	Change Percentage	−4.13 (−8.22–5.89)	−4.58 (−13.96–2.69)	*p* = 0.619 ^a^ r = −0.08

^a^: Mann-Whitney U test was used. EM: Multimodal exercise. EV: Vestibular exercise. Q1; Q3: First and third quartiles. r = Rosenthal’s “r”.

## Data Availability

The data presented in this study are available on request from the corresponding author.
